# Answering questions about consciousness by modeling perception as covert behavior

**DOI:** 10.3389/fpsyg.2015.00803

**Published:** 2015-06-16

**Authors:** Gustav Markkula

**Affiliations:** Adaptive Systems Group, Division of Vehicle Engineering and Autonomous Systems, Department of Applied Mechanics, Chalmers University of TechnologyGothenburg, Sweden

**Keywords:** neural correlates of consciousness, first-person subjective experience, perception, action selection, covert behavior, narrative behavior, heterophenomenology

## Abstract

Two main open questions in current consciousness research concern (i) the neural correlates of consciousness (NCC) and (ii) the relationship between neural activity and first-person, subjective experience. Here, possible answers are sketched for both of these, by means of a model-based analysis of what is required for one to admit having a conscious experience. To this end, a model is proposed that allows reasoning, albeit necessarily in a simplistic manner, about all of the so called “easy problems” of consciousness, from discrimination of stimuli to control of behavior and language. First, it is argued that current neuroscientific knowledge supports the view of perception and action selection as two examples of the same basic phenomenon, such that one can meaningfully refer to neuronal activations involved in perception as covert behavior. Building on existing neuroscientific and psychological models, a narrative behavior model is proposed, outlining how the brain selects covert (and sometimes overt) behaviors to construct a complex, multi-level narrative about what it is like to be the individual in question. It is hypothesized that we tend to admit a conscious experience of X if, at the time of judging consciousness, we find ourselves acceptably capable of performing narrative behavior describing X. It is argued that the proposed account reconciles seemingly conflicting empirical results, previously presented as evidence for competing theories of consciousness, and suggests that well-defined, experiment-independent NCCs are unlikely to exist. Finally, an analysis is made of what the modeled narrative behavior machinery is and is not capable of. It is discussed how an organism endowed with such a machinery could, from its first-person perspective, come to adopt notions such as “subjective experience,” and of there being “hard problems,” and “explanatory gaps” to be addressed in order to understand consciousness.

## 1. Introduction

The philosophical and scientific discussion of consciousness is age-old, but has also increased notably in intensity and level of detail during the last few decades, not least thanks to advances in the neurosciences. Many would argue that achieving a better understanding of consciousness, of how and why and under what circumstances it occurs, would be a fundamental step forward in our understanding of what it means to be human. Also, practical and ethical motivations can be raised, for example relating to assessing the degree of consciousness of human patients in comatose states, animals, or even man-built machines such as robots.

However, despite recent empirical and theoretical progress, many questions about consciousness remain hotly debated. One major such debate concerns how to define the *neural correlates of consciousness* (NCC), the “minimal neuronal mechanisms jointly sufficient for any one specific conscious percept” (Tononi and Koch, 2008, p. 239). A common approach to searching for the NCC has been the *contrastive* method (Baars, 1988), where brain activity during consciousness, for example of a visual stimulus, is contrasted with brain activity where consciousness is deemed to be lacking, generally because of a missing *subjective report*, for example a button press, in response to the stimulus. Many experiments of this type have been carried out (see e.g., Leopold and Logothetis, 1996; Srinivasan et al., 1999; Sergent et al., 2005; Lau and Passingham, 2006; Koivisto and Revonsuo, 2010; Pitts et al., 2012; Li et al., 2014), but variations in experimental paradigms, brain imaging techniques, and analysis methods have engendered seemingly conflicting results, which have been taken to support competing, markedly different hypotheses regarding the nature of the NCC (e.g., Tononi, 2004, 2012; Dehaene et al., 2006, 2014; Lamme, 2006, 2010; Block, 2009; Lau and Rosenthal, 2011).

One possible reason for the lack of consensus regarding the NCC could be that researchers have thus far not taken sufficient care to separate the brain activity involved in the actual conscious awareness (the *NCC proper*) from brain activity corresponding to prerequisites for or consequences of that awareness (Aru et al., 2012; de Graaf et al., 2012). However, some researchers and philosophers argue that even if neuroscientists could reach perfect agreement on the NCC, there would still remain an *explanatory gap* (Levine, 1983) regarding “why the neural basis of an experience is the neural basis of that experience rather than another experience or no experience at all” (Block, 2009, p. 1111), and that explaining a mere function or ability such as discrimination and reporting of a stimulus does not solve the *hard problem*: explaining the *first-person, subjective experience* of that stimulus (Chalmers, 1995). This allegedly unexplained aspect of consciousness is sometimes referred to as the *qualia*, the “raw feels,” such as “the characteristic experience of tasting a lemon, smelling a rose, hearing a loud noise or seeing the sky” (Jackson, 1982, p. 127), the “what it is like” (Nagel, 1974).

Whether or not there is such an explanatory gap to be bridged, or equivalently such a hard problem to be solved, is the topic of another major debate in contemporary writing on consciousness. Many have argued that these gaps and problems are illusions, arising from dualistic thinking or otherwise misguided intuitions (e.g., Dennett, 1991; O'Regan and Noë, 2001; Blackmore, 2012), and that something like qualia, such as discussed by the authors cited above, cannot exist (Dennett, 1988), at least not within the scope of science (Cohen and Dennett, 2011). Dennett (1991) has suggested what he calls the *heterophenomenological* approach to consciousness, whereby rather than asking questions like “why do we have qualia?,” which implicitly assume that first-person accounts of consciousness are *true*, we should ask questions like “why do we *say* that we have qualia?.” Blackmore (2012, 2015) has argued that even the very search for NCC bears the mark of dualistic thinking and is therefore bound for failure. O'Regan and Noë (2001) also deny the existence of qualia, and have proposed a behavior-oriented account of consciousness, whereby conscious experience amounts to active and task-oriented prediction of how sensory inputs will change with motor actions: “It is confused to think of the qualitative character of experience in terms of the *occurrence* of something (whether in the mind or brain). Experience is something we do and its qualitative features are aspects of this activity” (O'Regan and Noë, 2001 p. 960). This way of talking about consciousness comes rather close to the behaviorist (Skinner, 1984) and behavior analysis (Pierce and Cheney, 2004) traditions.

This paper aims to contribute to the two debates regarding (i) the NCC and (ii) first-person subjective consciousness, with an argument that is inspired from and related to those of Dennett, Blackmore, O'Regan, Noë, and others. The main means for extending beyond these previous contributions will be a model that, while simplistic, allows structured reasoning about both perception and consequent overt behavior, for example in terms of subjective reports about perceptions. A foundational observation behind this *narrative behavior model* is that current neuroscience construes both perception and selection of overt actions as rather similar in terms of the underlying neural mechanisms. The model therefore also helps clarify what one could mean by talking about perception as a form of behavior.

It will then be illustrated how the narrative behavior model can be put to use in a heterophenomenological approach to consciousness: It will be proposed that we tend to say that we are having a conscious experience of *X* precisely when we attempt to exhibit a covert narrative behavior describing *X*, and find ourselves capable of doing so. This proposal will be tested against some existing empirical findings from contrastive experiments, previously used to support competing hypotheses about the NCC, and it will be argued that apparent conflicts between these findings are resolved by the proposed account of consciousness. Specifically, the proposed account predicts that there need not be any well-defined, paradigm-independent NCCs to be found at the putative moment of conscious experience, and that any (approximate) common neural denominators should rather be sought around the moment of *judging* consciousness.

Finally, it will be argued that an organism functioning in accordance with the narrative behavior model would, given its narrative abilities and inabilities, have good reasons for thinking about its own “subjective experience” as something rather astonishing, enigmatic, and non-physical, such that the narrative behavior model could help explain the human tendency for postulating explanatory gaps and hard problems.

## 2. Shared brain mechanisms in action selection and perception

**Figure 1:**
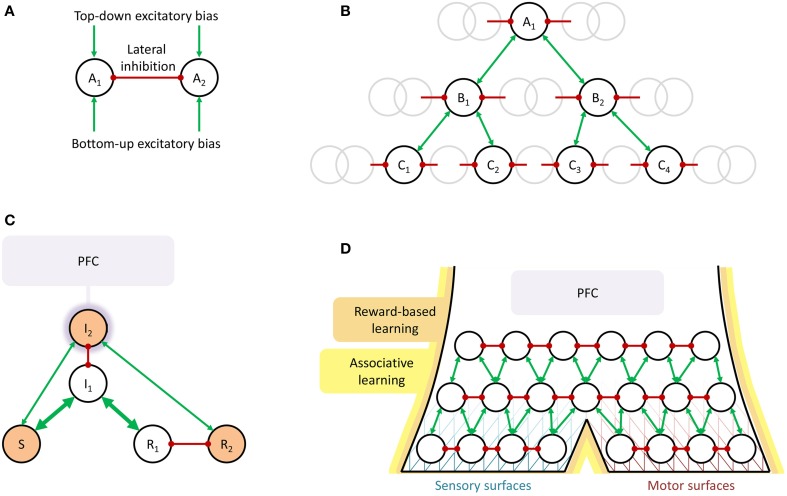
**Schematic illustrations of the suggested shared neural mechanisms underlying perception and selection of overt action**. **(A)** Biased competition. In perception, *A*_1_ and *A*_2_ could for example be CAT and LION, or the two alternative ways to perceive a Necker cube. In action selection, they could be LOOK AT ROAD AHEAD and LOOK AT CELL PHONE or MAKE TEA and DO NOT MAKE TEA. Typically, the nodes are also modeled as self-excitatory (Schöner et al., 1997; Deco and Rolls, 2004, 2005; Johnson et al., 2008), an aspect that will be left implicit throughout this paper. **(B)** Converging hierarchy, with the gray circles representing surrounding nodes not immediately involved in the current example. In perception, *A*_1_ could be LION, deduced from intermediate-level perceptual representations in the *B_i_* (speculatively, for example CAT-LIKE, LARGE SIZE, SAND-COLORED, etc.), in turn deduced from the *C_i_*, low-level perceptual representations of edges, colors, movement, etc. In action selection, *A*_1_ could be MOVE HAND TO GRASP KETTLE, *B*_1_ = EXTEND ARM, *B*_2_ = OPEN HAND, and the *C_i_* low-level motor commands to achieve these movements. An example where some or all actions cannot be performed simultaneously, thus inhibit each other, and must be performed sequentially or interleaved, could be *A*_1_ = MAKE TEA, *B*_1_ = BOIL WATER, *B*_2_ = ADD TEA-BAG TO CUP, *C*_1_ = FILL POT WITH WATER, *C*_2_ = PUT POT ON STOVE, *C*_3_ = GRASP TEA-BAG, *C*_4_ = PUT TEA-BAG IN CUP, and so on. **(C)** Guided activation. Prefrontal cortex (PFC) provides top-down bias resulting in activation (the orange shading of the nodes) of a non-routine mapping from stimulus *S* to response *R*_2_, rather than the stronger (the thicker arrows), routine mapping from *S* to *R*_1_. **(D)** Summary overview of shared mechanisms for perception and action selection. Biased competition in a converging hierarchy, growing from reward-based and associative learning, with PFC providing additional top-down bias when needed. The line pattern behind the nodes is intended to illustrate the idea that closer to the sensory and motor surfaces (receptors and effectors) the functional hierarchy can be traced more clearly in the neuronal hierarchy, whereas further away from these surfaces, there can be considerable neural overlap between separate functional nodes. Note the similarity to the model of for example Fuster (2000).

It is not common to talk about (i) selection of overt actions and (ii) perception, as in discrimination, classification, and to some extent interpretation of sensory stimuli, as being two examples of the same basic phenomenon. However, they have often been modeled quite similarly. This is especially clear in cases where the same mathematical model has been used for both. One such example is available from Deco and Rolls (2004, 2005), who have used a framework based on the concept of *biased competition* (see Figure 1A) to recreate a number of both more perception-oriented and more action-oriented experimental paradigms. In their simulations, pools of neurons, encoding for example a stimulus classification (i.e., perception) or a motor action (i.e., action selection), compete for activation by *lateral inhibition* between pools, *biased* by external excitation either *bottom-up*, e.g., from sensory input to which the pools are tuned, or *top-down*, e.g., signifying that what the pools are encoding (the stimulus classification or the motor action) is requested in the current task context. Using this type of model, it has been possible to explain various brain activation phenomena observed in object recognition paradigms (Deco and Rolls, 2004) and to reproduce observed overt behavior in paradigms with flexible stimulus-response mappings (Deco and Rolls, 2005). Zylberberg et al. (2010) have studied a similar computational model, integrating both elements of perception and action selection into one and the same neural simulation. Another modeling framework that has been used to explain and predict empirical observations both in visual perception and selection of motor actions is *dynamical field theory* (DFT), which also relies heavily on the idea of lateral inhibition between competing percepts or overt behaviors (Schöner et al., 1997; Johnson et al., 2008). See also the model of attention selection proposed by Engström (2008). Engström did not emphasize similarity between perception and action selection, but his model provided the starting point for what is being sketched here.

The basic idea of biased competition arose from the study of visual attention (Desimone and Duncan, 1995), but has since found empirical corroboration as an actual brain mechanism not only in sensory processing of visual (Kastner et al., 1998; Bichot et al., 2005), auditory, and tactile stimuli ((Porcu et al., 2014), but indeed also in selection of overt motor actions (Pastor-Bernier and Cisek, 2011). Thus, behavioral and neural data align to suggest that biased competition may be an important part of both how we settle on what percepts to extract from our sensory input, and how we settle on what overt actions to perform.

When talking about bias to these competitions coming “top-down” or “bottom-up,” a logical next step is to consider what, in the brain, is sending these biasing signals. One possible answer, attractive in its parsimony, would be: other competitions, at other levels in a *hierarchy*. Indeed, in visual object recognition, a converging hierarchical brain organization, as illustrated in Figure 1B, is part of the textbook description (see e.g., Bear et al.,^2001^), with neurons in early visual areas in occipital cortex responding for example to edges of specific orientations at specific spatial locations in the visual field, and neurons in later areas along the ventral pathway down onto the temporal cortex responding to increasingly complex features or objects, with increasingly overlapping spatial receptive fields. (This hierarchical progression, from V1 to IT, is also part of the abovementioned object recognition model of Deco and Rolls, 2004).

In motor control, the view of behavior as hierarchically organized, with goals and performance being represented and controlled at different hierarchical levels, goes back at least to Bernstein in the 1940s (Latash and Latash, 1994), and functional models of behavior built on this type of assumption have been able to generate complex behaviors with concurrent goals at different time scales (e.g., tea-making; cf. Figure 1B), while at the same time reproducing typical mistakes observed during such behavior (Cooper and Shallice, 2000, 2006; Crump and Logan, 2010). In line with this type of model, a single pointwise electrical stimulation of primary motor cortex can trigger seemingly goal-directed movements, such as reaching for a specific location in space ((Graziano et al., 2005; Graziano and Aflalo, 2007), interpretable as low-level *chunks* or *schemata* (Norman and Shallice, 1986) for use in the construction of more complex, compound overt activity. Neuronal activity in more rostral areas of frontal cortex, including prefrontal cortex (PFC), have been repeatedly shown to correlate with maintenance of goals for and control of such higher-level activity, with signs of hierarchical neuronal organization (Badre and D'Esposito, 2009), but the details of how this organization implements selection and control of actions are far from settled. It has been argued that rather than assuming an exact correspondence between neuronal hierarchies and task hierarchies, higher neuronal hierarchical levels could in general encode task goal representations that are to be sustained over longer time scales (Uithol et al., 2012).

Indeed, the idea of PFC being involved in sustaining neuronal activation over time has a long tradition, and also here both perception and action selection fit nicely within the same general framework, of PFC coming into play when something has to be kept active which could not otherwise sustain itself. Typical examples include *working memory*, for planned motor actions or perceptual representations to be maintained without any bottom-up sensory input (Fuster, 2000; Curtis and D'Esposito, 2003), as well as non-routine mappings between stimulus and response (Miller and Cohen, 2001; Deco and Rolls, 2005), see Figure 1C. One hypothesis is that the to-be-sustained information or sensorimotor pathways themselves reside in more posterior brain networks, but that they need excitation from PFC to achieve sustained activity (Miller and Cohen, 2001; Curtis and D'Esposito, 2003; Postle, 2006). In line with this idea, it has for example been shown that a classifier trained to decode from visual cortex fMRI data what visual stimulus a subject is seeing can also decode what stimulus is being kept in working memory (Harrison and Tong, 2009), clearly suggesting that the neural activity encoding a visual stimulus in working memory is to some extent the same as when actually seeing the stimulus. Within the context of visual perception, the dorsolateral region of PFC (DLPFC), in the middle frontal gyrus, has repeatedly been implicated in paradigms where subjects make an effort to select and sustain a certain visual percept, for example during periods of no visual input or distracting inputs (Fuster, 2000; Curtis and D'Esposito, 2003; Feredoes et al., 2011), in situations with ambiguous, bistable stimuli (Raz et al., 2007; de Graaf et al., 2011), or in mental imagery (Ishai et al., 2000; Schlegel et al., 2013).

Finally, there are similarities between perception and overt action also when it comes to learning. *Rewards* lead to the adoption of those overt behaviors and stimulus-response mappings that are beneficial to the individual in its given environment. Important here are loops between the cortex and the basal ganglia, where reward-dependent release of dopamine in the striatum determines neural plasticity (Yin et al., 2008; Gurney et al., 2015), and notably such reward-gated corticostriatal loops seem equally important when learning perceptual classification of stimuli into categories (Seger and Miller, 2010; Cantwell et al., 2015). Learning can also be of a Hebbian, *associative* form, where neurons that have been concurrently active strengthen their mutual connections. Such learning, with the hippocampus as an important structure both for storage and for consolidation of connections elsewhere, has been well-studied when it comes to long-term memory of sensory events (i.e., *episodic memory*; Henke 2010) but also seems to occur in learning of motor sequences (Schendan et al., 2003; Albouy et al., 2008). Additionally, cortico-cortical associative learning has been suggested as a mechanism for late-stage consolidation of acquired abilities, both within perception (Ashby, 2013) and motor control (Ashby et al., 2010).

In sum, a picture emerges in which the brain seems to be controlling both what is perceived and what is overtly done in roughly the same ways, schematically summarized in Figure 1D. Specific activation patterns encoding specific percepts or overt actions entering in biased competition with other, incompatible percepts and actions, in a converging functional hierarchy which is at least at its lower levels reflected in a hierarchical neuronal organization. The nodes in the functional hierarchy and their interconnections arise from reward-based and associative learning, and an important role of PFC is to support sustained maintenance of node activation when needed.

This is, of course, an extremely simplified description of brain function, and there are sure to be many finer points where perception and action selection differ in their implementation. In general, however, it makes sense that the brain might capitalize on the same mechanisms to optimize both motor and perceptual activity; both are equally crucial for ensuring the organism's survival and reproduction. Most overt actions depend on perception (perceive the lion, then sneak away from it), similarly to how some overt actions depend on other overt actions (get the apple out of the tree, then eat it). Furthermore, for the present purposes the shared framework outlined above seems useful in at least three ways:

(i) It allows the suggestion that activation patterns (nodes in the functional hierarchy) could be referred to with a shared term, regardless of whether they encode percepts or overt actions. Based on the idea that both are learned and selected to optimize for survival and reproduction, the shared term that will be adopted here is *behavior*. Thus, an individual can be regarded as capable of both *overt behavior*, when exhibiting externally observable body movement, and *covert behavior*, when exhibiting neuronal activation encoding percepts or overt actions, that may or may not be immediately associated with body movement. One of the aims of this paper is to show that using the term behavior in this way establishes a mindset that can lead to more fruitful discussions of consciousness. (ii) The framework clarifies in more operational detail what one might mean by claiming perception to be a type of behavior, as some authors have (Skinner, 1984; O'Regan and Noë, 2001; Pierce and Cheney, 2004). (iii) It will serve as the basis for the narrative behavior model to be sketched in the next section.

## 3. A model of selection of covert and overt narrative behavior

Now, the following is defined:

***Narrative behavior**: A behavior which can be interpreted, by a third-party observer, as a communication, to one or more recipients, of some aspect of how the behaving individual is currently classifying the state of the world (including the individual's own body)*.

Such narrative behavior can be overt, such as when an individual speaks (or signs, or writes) statements like “my foot hurts” or “there is a lion in the bushes over there,” typically with one or more other individuals as the recipients. Narrative behavior can also be covert, in which case the recipients are simply all the parts of the individual's brain to which the involved neurons project. It should be noted that it is not required that these recipients “understand” what is being communicated, in the sense of understanding as we attribute it to entire individuals. In Dennett's (1978) terms, a covert narrative behavior and its reception take place on the *subpersonal* level, whereas “understanding” is a phenomenon on the *personal* level.

**Figure 2:**
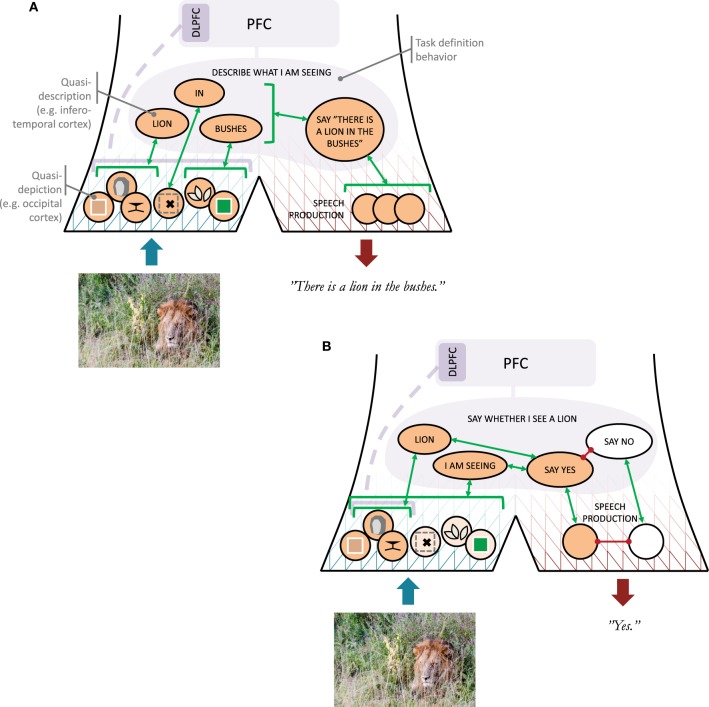
**Illustration of the narrative behavior model**. **(A)** Roles of quasi-depictions and quasi-descriptions (with rough cortical locations indicated for the visual modality) as well as a task definition behavior, in an example situation where a subject is to provide a verbal description of what he or she is seeing. An arrow to a bracket indicates a one-to-many association. The dashed line from DLPFC (dorsolateral prefrontal cortex) to the quasi-depictions indicates uncertainty whether or not DLPFC involvement would be needed in this situation. **(B)** As above, but now the subject has been asked to say whether or not he or she sees the lion.

Then, by the definitions adopted in this paper, perception is clearly a case of covert narrative behavior, or, considering what was said in the previous section, typically an entire set of concurrent covert narrative behaviors, on different hierarchical levels. For simplicity, I will separate this hierarchy into one lower and one higher level, which I will refer to as *quasi-depiction* and *quasi-description*, respectively (Kosslyn, 2005, also employed the depiction-description distinction). Consider an example, illustrated in Figure 2A: If I foveate a lion hiding in bushes, I will exhibit low-level narrative behaviors communicating about edges, colors, and orientations corresponding to various features of the lion, the bushes, and so on. Taken together, these narrative behaviors could be likened to a “depicting” of the visual scene being processed, but the qualifier “quasi” in quasi-depiction intends to emphasize that the purpose of these low-level communications is not for them to be assembled into a “full picture” somewhere else in the brain. The totality of all concurrent quasi-depictions can in itself be considered a kind of “full picture,” but it is not needed for what will follow here that this totality is ever received somewhere and integrated into something less parallel (cf. Dennett, 1991, e.g., p. 135).

At higher hierarchical levels, the covert narrative behaviors communicating about the scene with the lion will start becoming less like depictions, and more like verbal descriptions. This could amount to classification of quasi-depictions into learned *concepts*, such as LION or BUSHES. It is further assumed here that also a concept such as IN can be communicated at this quasi-descriptive level (e.g., in the form of an *image schema*; Lakoff 1987; Rohrer 2006), allowing composition of narratives like LION IN BUSHES. Here, the qualifier “quasi” intends to emphasize that while these covert narrative behaviors are language-like, in the sense of operating with concepts and some compositionality, they are not fully articulated verbal descriptions, expressed in overt or covert speech. For lack of a better means, quasi-descriptions will be referred to here using capitalized words or sentences, and they could be understood as related to the *constructions* hypothesized in some cognitive linguistics approaches (Goldberg, 2003; Feldman, 2006), but it is not necessarily the case that quasi-descriptions always have an easily identified counterpart in language. Furthermore, when such counterparts in language do exist, such as the words “lion” and “bushes,” it should be noted that in a convergent hierarchy of narrative behaviors both of these words may well be associated with a *range* of quasi-descriptions, such as for example BUSHES(1), BUSHES(2), and so on, of which the sensory stimuli at hand will activate only one (or perhaps a few?). For this reason, the word “bushes” will be an imperfect approximation of the activated BUSHES(*i*), just as BUSHES(*i*) will be an imperfect approximation of the specific quasi-depictions that triggered it.

It is proposed here that quasi-descriptive narratives account for some types of *perceptual completion* phenomena (Pessoa et al., 1998), such as in Dennett's (1991, 1992) oft-mentioned example with a wallpaper of identical images of Marilyn Monroe. When glancing upon such a wallpaper, only one or a few portraits will be sensed with high resolution at the fovea; the non-foveated portraits will not be distinguishable from colored blobs, yet the “perceptual experience” is that of an entire wall of identical Marilyns. Dennett argued that the brain should not need to “fill in” the remaining Marilyns anywhere in the brain; it should be enough for the brain to just draw a conclusion on a symbolic level that, here, can be understood as a quasi-description A WALL FULL OF MARILYNS, and that this is enough to account for the “perceptual experience” in question. Experimentally, perceptual completion phenomena have in some cases been found to be associated with actual neural filling-in (e.g., De Weerd et al., 1995), in other cases not (e.g., von der Heydt et al., 2003), and in some cases a neural filling-in at lower levels seems to depend on feedback from higher levels (Sterzer et al., 2006), interpretable as feedback from quasi-description to quasi-depiction.

To finalize the example with the lion (Figure 2A), based on a quasi-description such as LION IN BUSHES, I may come to articulate a verbal description of what I see, either overtly or covertly (in terms of brain function, the two may be rather closely related; Scott et al., 2013). The reason why I might select to generate an overt description is at least in this specific case most probably reward-related. For example, someone may have asked me to describe what I am currently seeing in a photo, and I thus seek the social reward of complying with instruction. Alternatively, I may really be in person on the savannah together with someone, such that there is a clear survival aspect to communicating my observation. In either case, the process can be understood as a high-level behavior DESCRIBE WHAT I AM SEEING having become activated, possibly implemented in PFC or maintained in sustained activity with excitation from PFC. This type of behavior will be referred to here as a *task definition behavior*. Considered in isolation, this is not a narrative behavior, but the overt speech act that it causes (“There is a lion in the bushes”) clearly is.

## 4. A heterophenomenological analysis of consciousness: when do we tend to say that we have a conscious experience?

If, after giving my overt verbal report about the lion in the bushes, someone asks me whether I have “a conscious experience of seeing the lion” or “a conscious visual experience of the lion,” I am likely to answer in the positive. Why is this? In this section, the narrative behavior model will be applied in a heterophenomenological analysis, to begin with of the the special case of “seeing.” In practice, this will amount to introducing a set of hypotheses on what is required for an individual to admit to be seeing under various circumstances. These proposed hypotheses, in effect operational definitions of what “seeing” means, will then be generalized across sensory modalities, to consciousness in general.

If someone asks me “do you see the lion?,” and I answer yes, this has to depend on some acquired concept of mine of what it means to “see.” In the terms of the model introduced above, just as one can hypothesize quasi-descriptions LION and BUSHES, one can hypothesize a quasi-description I AM SEEING, which is excited whenever I engage in covert narrative behavior of a visual nature. Specifically, the following operational definition is proposed:

***“Seeing”**: We tend to say that we see X when we attempt to describe X by means of covert narrative behaviors providing visual detail about X, and find ourselves acceptably capable of doing so*.

This needs clarification. As illustrated in Figure 2B, what is proposed is that a question “do you see the lion?” activates a task definition behavior that we can label SAY WHETHER I SEE A LION, which involves (i) a singling out of the quasi-descriptions I AM SEEING and LION, (ii) an attempt to activate, top-down, quasi-depictions that associate with these quasi-descriptions, and (iii) a mapping from a sufficient concurrent activation of I AM SEEING and LION to a SAY YES behavior. The top-down excitation of narrative behaviors that associate both with I AM SEEING and LION is thus an example of an “attempt to describe *X* by means of covert narrative behaviors providing visual detail about X.” In line with what has been said above, this attempt is suggested to require PFC activation, to keep the SAY WHETHER I SEE A LION complex active, and perhaps also DLPFC to single out and bias further specific quasi-depictions directly. If I am already looking at the lion when the question is asked, some of the lion-associated visual quasi-depictions may already be active, but if the top-down excitation triggered by the question is successful, the total amount of such activation will increase, resulting in enough bottom-up excitation to push the SAY WHETHER I SEE A LION complex to a threshold of activation where the SAY YES behavior is generated. The reaching of this threshold is thus an example of me “finding myself acceptably capable” of generating the “covert narrative behaviors providing visual detail about *X*.”

Regarding the “narrative behaviors providing visual detail,” assumed to excite I AM SEEING, which are these? One answer could be “any narrative behavior that is implemented along the brain's visual pathways.” However, as suggested by O'Regan and Noë (2001), a more accurate account might instead link I AM SEEING to narrative behaviors obeying *sensorimotor contingencies* of a visual type; i.e., narrative behaviors that change in typical, predictable ways in response to for example eye movements.

The above account of what I do before confirming to be seeing the lion clarifies further how seeing could be likened to an overt behavior, since my judgments of my own attempts at overt behavior can be modeled in much the same way. If I am monitoring my own success at whistling, for example, this can be described in the present model as attempting to activate a quasi-description I AM WHISTLING, associated with specific quasi-depictions that encode the auditory and perhaps lip and tongue-related somatic sensations of successful whistling. (There is an illustration of this example in **Figure 5A**.) The less my exhaled air sounds like hissing and instead like a tone, the more the whistling-related quasi-depictions will become activated, the more activated I AM WHISTLING will be, and the more likely I will be to admit success. In other words, admitting to seeing the lion requires producing enough of the neuronal activations that associate with I AM SEEING and LION, just as admitting to whistling requires producing enough of the neuronal activations that associate with I AM WHISTLING. The major difference is that only in the latter case are there concurrent externally observable effects, such that a third party can also provide judgment of success or failure.

What if there is a delay between a stimulus and a question regarding my perception of it? (This is often the case in contrastive experiments on the NCC, as will be exemplified further below.) Suppose, for example, that I am only briefly shown the photo with the hiding lion, and then asked the question: “Did you see the lion?” For this type of case, a modification to the operational definition above is proposed, as follows:

***“Just having seen”**: We tend to say that we just saw X when we attempt to describe X by means of covert narrative behaviors providing visual detail about X, and, with the support of short-term visual memory, find ourselves acceptably capable of doing so*.

In other words, it is suggested here that admitting to just having seen the lion also requires that I find myself acceptably capable of covert narratives describing the lion, the important difference being that now these covert narrative behaviors have to be activated without bottom-up input. As has been discussed previously, such top-down-only maintenance of recently active covert visual narratives seems to require DLPFC involvement. Another aspect to note is that without bottom-up input, the threshold for what is an acceptable level of visual detail might very well be lower, perhaps more so the longer the duration between stimulus and question.

If this temporal duration starts outgrowing the “just having seen” denomination, why not even with other stimuli in between (e.g., other photos, without any lions in them), a positive answer to the question “did you see the lion?” could be hypothesized to require only very imprecise reactivated covert narratives, perhaps even at a more quasi-descriptive than quasi-depictive level, i.e., simply LION without any details. Also, rather than (or in addition to) being dependent on structures such as DLPFC, one could hypothesize that such reactivation relies on brain structures that have been implicated in longer-term memory recall, in the form of episodic recollection or familiarity (Yonelinas et al., 2005). The following operational definition attempts to accommodate all of what has been said so far into one, more general account of “seeing:”

***Current or historical “seeing”**: We tend to say that we see X (or that we saw X) when we attempt to describe X by means of covert narrative behaviors providing visual detail about X, and, with the support of visual input (or visual memory at short or long time scales), find ourselves acceptably capable of doing so (probably with lower requirements for more historical X)*.

What about “consciousness,” then? It is suggested here that (i) the above operational definitions of “seeing” can be modified to definitions of “hearing,” “smelling,” “feeling,” and so on, simply by replacing “visual detail” by “auditory detail,” “olfactory detail,” “tactile/proprioceptive/nociceptive/… detail,” and so on, and (ii) one will tend to admit “having a conscious experience” precisely in those cases where one admits to one or more of “seeing” or “hearing” or “smelling” or “feeling,” etc. Thus, in summary form, one arrives at the following proposed operational definition of “consciousness,” i.e., the following hypothesis on what it takes to admit consciousness:

***Current or historical “consciousness”**: We tend to say that we have a conscious experience of X (or that we had a conscious experience of X) when we attempt to describe X by means of covert narrative behaviors providing perceptual, as in visual/auditory/olfactory/etc., detail about X, and, with the support of current sensory input (or memory of previous sensory events at short or long time scales) find ourselves acceptably capable of doing so (probably with lower requirements for more historical X)*.

The main difference between this account of consciousness and most existing theories, is that it shifts the focus from around the point in time *T*_x_ when *X* took place, to the time *T*_j_ when we judge whether we are (for *T*_j_ = *T*_x_) or were (*T*_j_ > *T*_x_) conscious of *X*. Under this view, there is no need to assume that there are any neural correlates of consciousness in the way this term is normally understood, i.e., there need not be any well-defined, precise common denominator for all brain states that will engender a concurrent or later report of consciousness. The proposed common denominator lies instead in the moment of introspection, where consciousness of present or past events will be subjectively judged based on the ability for covert narrative about them. However, even this common denominator could be rather imprecise, given that the criterion for judgment may not be constant, but instead relaxed over time, with variations also in which brain mechanisms related to short or long term memory are involved.

These differences between the proposed account and others will be considered in more detail further below. First, however, the proposed account will be tested against some existing empirical data.

## 5. Application to selected empirical results

Here, three experiments from the literature will be described and analyzed. These specific experiments have been chosen because they have been previously used in arguments for competing, rather different theories of consciousness and the NCC (the theories as such will be discussed in the next section).

**Figure 3:**
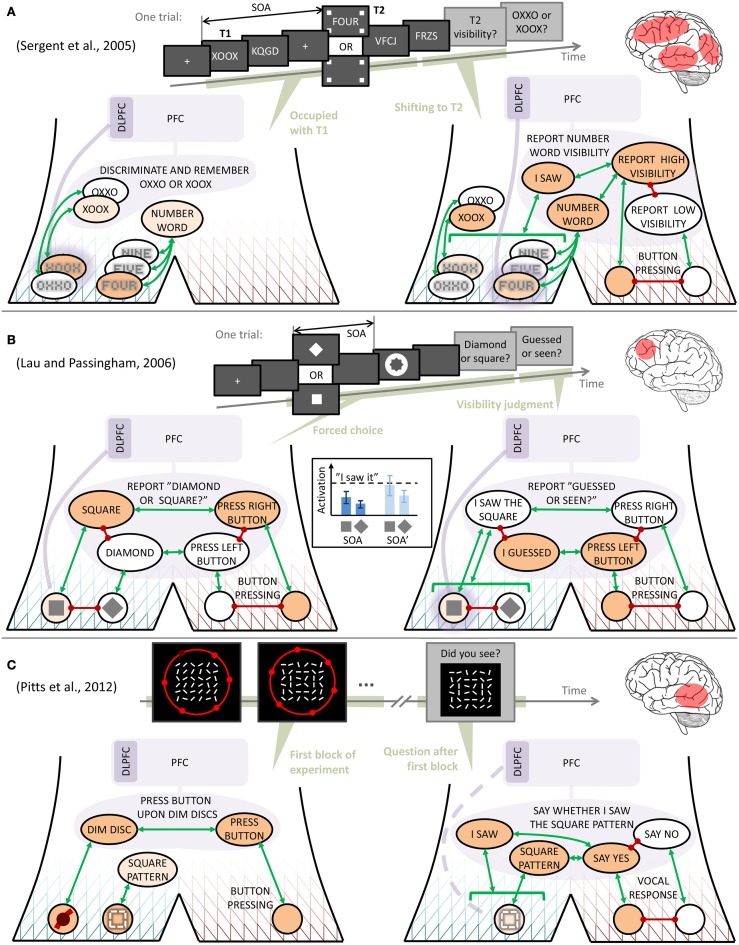
**Interpretation of findings from three contrastive experiments on visual consciousness**. Symbols as in Figure 2, except in **(A)**, where, to avoid clutter, mutual inhibition is in some cases indicated by stacking behaviors on top of each other, rather than with red lines. The inset in **(B)** shows one possible way in which the relationship between quasi-depiction strengths might yield similar discrimination performance across two stimulus onset asynchronies (SOAs), yet differing reports of discrimination confidence. In each of the panels **(A–C)**, a schematic illustration is also given of the experimental paradigm, as well as a rough indication of the main contrasts in brain activation that the original authors found between stimuli reported as seen rather than unseen.

Consider, first, the study by Sergent et al. (2005), schematically illustrated in Figure 3A, where subjects in an *attentional blink* paradigm reported a characteristic low visibility of a target stimulus T2 (a four-letter number word), when simultaneously performing a discrimination task on a target stimulus T1 (one of the two strings OXXO or XOOX), presented a *stimulus onset asynchrony* (SOA) interval earlier. As also illustrated in the figure, using the narrative behavior model this phenomenon can be understood as brain structures needed for establishing a task definition behavior (including PFC) and top-down biasing of quasi-depictions (DLPFC), becoming occupied with discriminating and storing T1 after its presentation. During this process, the T2 stimulus arrives and generates, bottom-up, some quasi-depiction and quasi-description, consistent with the early occipital and occipitotemporal activations that Sergent et al. (2005) observed for both seen and unseen T2. When the processing of T1 is complete, the brain reorganizes its activity to accommodate the task on T2, at which point the bottom-up activations for this stimulus may either have decayed too much, leading to a report of non-visibility, or remain strong enough to allow DLPFC-assisted maintenance, acceptable activation of NUMBER WORD and I SAW quasi-descriptions, and the consequent report of a visible T2. The latter sequence of events would then correspond to what Sergent et al. (2005) observed in the form of brain activations in temporal, frontal, cingulate, parietal, and occipital regions (roughly in that time order) for seen but not unseen T2. Note that this account of the attentional blink is very similar to several existing, more detailed models (Dehaene et al., 2003; Dux and Marois, 2009; Zylberberg et al., 2010).

Next, consider the observations from the *metacontrast masking* experiment of Lau and Passingham (2006, Figure 3B). These authors found two SOAs between a target visual stimulus (a square or a diamond) and a subsequent mask, at which discrimination performance was the same, yet with different reported subjective confidence in the discrimination. Using the narrative behavior model, the following interpretation can be made: First, the temporal proximity of target and mask makes unambiguous activation of the correct quasi-depiction difficult (presumably through lateral inhibition in early visual processing; Di Lollo et al., 2004), such that a possible dominance of the target quasi-depiction may be rather weak. However, since the subject has to make a forced choice of just one stimulus, the task definition behavior REPORT “DIAMOND OR SQUARE?,” has to converge to one of the two quasi-descriptions SQUARE or DIAMOND, and this competition is biased toward the stimulus that is more active than the other at the quasi-depictive level, even if weakly so. Similar discrimination performance at two different SOAs could then be understood as the two SOAs generating relationships between the quasi-depiction strengths that are functionally similar in this sense; for example as in the inset in Figure 3B, where the *relative* difference between quasi-depiction strength is preserved between SOAs.

Then, when reporting confidence in the discrimination just carried out, the subject sustains, by means of top-down DLPFC activation (that may well have been in place since the appearance of the stimulus), the quasi-depictive activation just selected, and judges whether it is strong enough for admitting to having seen the stimulus. If (i) this judgment instead depends on something more like the *absolute* strength of the selected quasi-depiction, as suggested by the dashed threshold in the inset in Figure 3B, and (ii) the two different SOAs identified by Lau and Passingham (2006) differed in this more absolute sense (for example because the different SOAs made certain attributes of the stimuli differentially salient; see Lollo, 2012), then one can understand how performance and confidence may dissociate in this paradigm. Furthermore, if assuming reciprocal connections between DLPFC and the quasi-depictions, this type of account also explains why 2006 found that DLPFC activation correlated with confidence but not with performance, as long as only higher *absolute* levels of quasi-depiction result in higher absolute levels of DLPFC activation. The interpretation presented here is also in line with the finding of Rounis et al. (2010), that TMS to DLPFC during this experimental paradigm reduces confidence ratings without affecting discrimination performance; this manipulation makes the subjects incapable of maintaining the quasi-depictions active until the time of judging confidence.

Finally, consider the experiment by Pitts et al. (2012, see Figure 3C), in which subjects were, in a first experimental block, to respond with a button press to discs in their peripheral visual field going dim, while fixating a centrally located large matrix of frequently reorienting line segments. Unbeknownst to the subjects, every now and then some of these line segments aligned to briefly form a square pattern (pattern exemplified in Figure 3C, but note that in this schematic illustration the pattern seems less conspicuous than in the actual stimuli used by Pitts et al.). After this first experimental block, subjects were shown a number of line matrix patterns, including the square, and asked whether they had seen them. Pitts et al. found that, when originally presented, the square pattern had caused a measurable early event-related potential, seemingly from occipital areas, in all subjects. However, in those subjects who afterwards reported having seen the square with some confidence, there was also an additional, later potential, seemingly from more temporally located parts of the brain. Signs of more frontal brain activations were only observed in subsequent experimental blocks, where the square pattern was made part of the subjects' task.

Figure 3C provides an interpretation: In the first block, the subjects complied with instruction by setting up and maintaining a task definition behavior that mapped dim discs to button presses. When the square pattern formed, all subjects quasi-depicted it (the early, occipital activation), but more semantic processing (the slightly later, more temporal activation), understandable as a quasi-description SQUARE PATTERN, occurred only in some subjects. Crucially, at this point the subjects had no reason to sustain the quasi-depiction, e.g., by engaging DLPFC, and discriminate or judge it. However, by associative learning, the repeated association of a specific quasi-depiction with the SQUARE PATTERN quasi-description left a memory trace, giving these subjects a greater chance of later reactivating the quasi-depiction, should this ever become needed. And indeed, the need arose when Pitts et al. questioned the subjects after the first block. As illustrated in the right part of Figure 3C, the subjects' response, at this point, about whether they had seen the square pattern earlier, can be understood as dependent on their ability to reactivate this quasi-depiction from memory. In line with what was said in the previous section, it could also be that this reactivation occurred at the quasi-descriptive level. The exact mechanisms of this recollection process are, however, not crucial here, as long as the reactivation is facilitated by the SQUARE PATTERN activation during the first experimental block.

Summarizing, the brain imaging contrasts for consciousness were in all these three experiments based on a subjective question of the type “did you see *X*?,” but the experiments by Sergent et al. (2005) and Lau and Passingham (2006) differed in that the latter authors also asked the question “what was *X*?,” and used it as a control to filter away discrimination-related brain activity that did not change with changing responses to the “did you see *X*?” question. On the other hand, in both these experiments this question on visibility was asked very shortly after presentation of *X*, and was expected by the subjects, meaning that the subjects could start engaging in introspective judgment even before the question was asked. According to the account proposed here, this early judgment is the reason why both experiments showed DLPFC activity in their observed neural correlates of later positive report. In contrast, the subjects of Pitts et al. (2012) did not expect the question on visibility, and the observed neural correlates therefore instead reflected such stimulus-triggered quasi-descriptive activity that was sufficient for a positive judgment once the question was asked, considerably later. Thus, all of these findings can be qualitatively understood using the model and hypotheses proposed here. As will be clear from the next section, previous authors have argued that one or the other experiment provides a more accurate test of consciousness. The account proposed here rather suggests that the different experiments simply engaged their subjects in self-judgment of consciousness in different ways.

## 6. Relation to some existing accounts of consciousness

Dehaene et al. (2003, 2006, 2014) have argued for a *global neuronal workspace* (GNW) theory of consciousness, originally suggested by Baars (1988). The widespread brain activation associated with reports of a visible T2 stimulus in an experiment such as that of Sergent et al. (2005, Figure 3A) is, according to this view, a signature of T2 processing entering the hypothesized GNW, an interconnected set of areas across the brain with the purpose of “flexible sharing of information throughout the cortex [for example to] route a selected stimulus through a series of non-routine information processing stages” (Dehaene et al., 2014, p. 79). A flexibly mapped, non-routine reaction to a stimulus, such as in the experiments discussed above, is assumed to require that the stimulus first becomes conscious by entering the GNW, making consciousness and the GNW the bottleneck behind phenomena like the attentional blink or the *psychological refractory period* (Marti et al., 2012). Indeed, behavioral and neuronal aspects of these phenomena have been well accounted for by computational modeling of such a central “routing” resource (Dehaene et al., 2003; Zylberberg et al., 2010). Put succinctly, in the GNW account, if I am to (flexibly, non-routinely) respond to a stimulus, I first need to “see” it consciously, and consciousness is a limited resource.

The model presented in this paper seems to be consistent with the experimental data and modeling work used to support the GNW hypothesis. What has been presented, here, as PFC-mediated flexible mappings between stimulus and response could be understood as identical to the flexible routing function attributed to the GNW, and could presumably be modeled precisely as by Zylberberg et al. (2010). The main difference lies in the assumption, by Dehaene and colleagues, that this type of central processing determines consciousness. According to the GNW hypothesis, as soon as there is central processing, consciousness (somehow) arises. According to the present view, since central processing of a stimulus *X* increases the amount of activation in the brain associating with *X*, perhaps including DLPFC-assisted fixation of quasi-depictions of *X* as part of the task at hand, such central processing would be associated with improved concurrent and later ability of activating covert narratives describing *X*. Therefore, central processing of *X* for flexible, non-routine behavior will be something like a *sufficient* condition for admitting consciousness of *X*, but not therefore a necessary condition, as for example suggested by the experiment by Pitts et al. (2012)^1^.

Lau and Rosenthal (2011) also argue against the GNW view, and emphasize that dissociations between performance in discriminating a stimulus and confidence in having seen it, as observed by Lau and Passingham (2006, Figure 3B) and Rounis et al. (2010), seem incompatible with the idea of a global workspace mediating both flexible responding and conscious perception. Instead, Lau and Rosenthal (2011) suggest that the DLPFC activity associated, in these experiments, with confidence in having seen the discriminated stimuli, reflects *metacognition* about the subjects' own mental states, in line with *higher-order* theories of consciousness. In these theories, phenomenal consciousness of *X* depends not on *first-order* mental representations of *X* (e.g., in early visual areas), but on higher-order representations, “that represent oneself as being in the relevant first-order mental states” (Lau and Rosenthal, 2011, p. 365; for an overview of alternative versions of higher-order theory, see Carruthers, 2011).

In one sense, the view presented here is clearly in line with the higher-order view. Here, it has been suggested that a subject will admit seeing or having seen a stimulus *X* when he or she succeeds in generating covert narratives providing visual detail about *X*, to the extent that a quasi-description I SEE *X* or I SAW *X* wins over its competitors (e.g., I DON'T SEE *X* or I GUESSED *X*). Such quasi-descriptions can clearly be regarded as metacognitive, representing the subject as being in the state of “seeing *X*” or “having seen *X*.” Furthermore, just as suggested by Lau and Rosenthal (2011), DLPFC has been implicated here as a necessary component in this metacognition, at least in the case of very recent historical *X*.

However, one can possibly discern an additional, unstated assumption in the reasoning of Lau and Rosenthal (2011), that is not a part of the framework proposed here: the view of conscious awareness as something that “arises” in the moment (“How does awareness arise?”, p. 366), with a well-defined, scientifically identifiable *correct answer* as to what the contents of conscious awareness are at a given point in time (“a second stage process […] determines *what enters awareness”*, p. 367; “In peripheral vision […] do we *actually consciously experience” vivid details of color and shape, or mistakenly think that we do[…]?*, p. 371; my italics in both cases)^2^. Differently put, this is the assumption that the concept of neural correlates of consciousness, as it is normally used, makes sense, something which could for example be formulated as follows:

***The NCC assumption**: When an individual is exposed to a stimulus X at a time T_x_, this may or may not give rise to a conscious experience C(X) of this stimulus at a time T_c_ = T_x_ + ϵ, where ϵ is some rather short duration, and if this conscious experience C(X) occurs, it is because of a specific, well-defined neural correlate of C(X) also occurring, in the brain, at time T_c_*.

The account proposed here does not assume that well-defined conscious experiences *C*(X) are continuously generated in or by the brain, for the subject to introspect and the neuroscientist to detect. Rather, it is proposed that consciousness is *only ever* subjectively judged, and that this judgment occurs in the moments of introspection or remembrance, or at requests of subjective reports. Thus, a possible divergence between what has been proposed here and higher-order theories could occur in the interpretation of an experiment such as that of Pitts et al. (2012). If a higher-order theorist contends that the subjects who reported having seen the square pattern in this experiment must have engaged in metacognition *when the pattern was presented* (i.e., at *T*_c_ ≈ *T*_x_), since these subjects were apparently “conscious” of the pattern, then that theorist is not in line with the present proposal (and must explain the apparent lack of frontal brain activity in response to the pattern). If, however, the higher-order theorist holds that the metacognition, e.g., in the form of a quasi-description I SAW THE SQUARE PATTERN, occurred at a later time *T*_j_, just after the time *T*_q_ *when the question was asked*, then that higher-order theory and the account presented here align.

Another type of interpretation of the experiment by Pitts et al. (2012) is that the more widespread and frontal brain activations observed in experiments such as those by Sergent et al. (2005) or Lau and Passingham (2006) reflect post-perceptual processes, for example to prepare or carry out a subjective report (Aru et al., 2012; Pitts et al., 2014). What has been said here partly aligns with this idea, but nuances it by suggesting that some frontal activations, not least in DLPFC, may be intrinsic and inescapable components of “seeing” a presently or very recently available stimulus, albeit not necessarily of “seeing” a stimulus that one is asked about only later.

**Figure 4:**
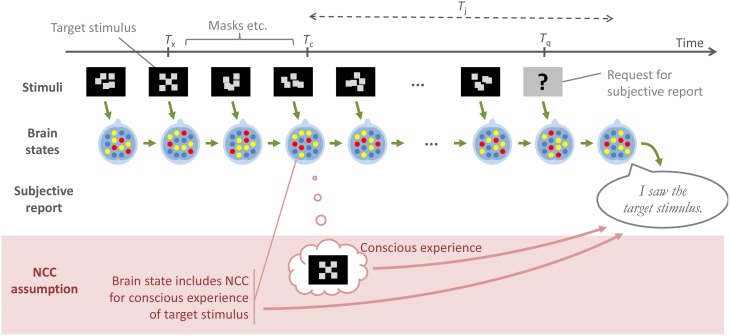
**A schematic illustration of a generic experimental paradigm, where a subject reports at time *T***_q_**, on a target stimulus that was presented at time *T***_x_. All of the contrastive experiments considered in this paper are of this general type. The green arrows in the figure denote a low-level, uncontroversial path of causation, from one stimulus and brain state to the next, and then to the overt subjective report. The lower part of the figure, and the red arrows, illustrate how authors operating under what has here been called *the NCC assumption* often reason as though a brain state at a putative moment *T*_c_ of conscious experience is what causes, and therefore should be sought in order to explain, the subjective report at *T*_q_. The account proposed here instead links this report to the outcome of a judgment of consciousness by the subject, at a time *T*_j_ which, depending on the exact experimental paradigm, may occur either in response to the stimulus, or in response to a question about the stimulus.

The NCC assumption, as defined above, and the type of reasoning it often inspires is further illustrated in Figure 4. The top half of the figure shows a low-level chain of causation that most would probably agree on: At each point in time, the state of the brain results from the immediately preceding brain state, combined with immediately preceding external stimuli. Furthermore, an overt behavior, such as a subjective report about a stimulus, is the result of a specific brain state immediately preceding the overt behavior. The bottom part of the figure illustrates how authors operating under the NCC assumption have in practice adopted a simplified view of this causation, whereby a subjective report in a contrastive experiment is construed as dependent not on the brain state following *T*_q_, and thus indirectly on the entire preceding sequence of stimuli and brain events, but instead only on whether or not an assumed NCC, with its associated conscious experience, was formed at *T*_c_.

There is nothing wrong in general with making this type of simplification. For example, within a given, fixed experimental paradigm there might well be a clearly identifiable difference at some *T*_c_ between brain states which do and do not engender a later report of consciousness, for instance because of covert introspective judgment carried out at *T*_c_, such that *T*_j_ = *T*_c_, or some other threshold effect occurring somewhere in the brain around this time. However, what is suggested here is that if the experimental paradigm is modified, the same identifiable difference in brain state at *T*_c_, or the same threshold effect, might no longer be what makes the difference for the report after *T*_q_.

In summary: Under the NCC assumption, it suffices to know the subject's current brain state and the NCC for the percept in question to know whether the subject is currently conscious of it. Under the view proposed here, there is no well-defined answer to the question of what a subject is conscious of at any one given time; one also has to specify (at least) when the subject will be asked about his or her perceptual experience, what other stimuli will be presented before that point in time, and whether the subject knows that the question is coming. This critique of the NCC assumption comes close to what has been argued by Blackmore (2012, 2015). It is also similar to Dennett's rejection of the idea of a “Cartesian Theater” in the brain, at which the information in conscious experience would come together for the benefit of… “whom? The Queen?” (Dennett, 1991, p. 255). In general, the present account seems to stay roughly within the theoretical perimeter circumscribed by Dennett's *multiple drafts* theory (Dennett, 1991) and his “fame in the brain” metaphor (Dennett, 2001), but what has been proposed here delimits these further with a more precise model and definitions.

There is compatibility and partial overlap also with the sensorimotor account of O'Regan and Noë (2001). It has already been pointed out that their view and the present one share the same emphasis on perception as behavior, and that the type of sensorimotor contingency that a specific quasi-depiction obeys could be (part of?) determining the subjective modality of that quasi-depiction, i.e., which of the quasi-descriptions I AM SEEING, I AM HEARING, etc. that the quasi-depiction tends to activate. In general, O'Regan and Noë's foundational concept of “mastery of the laws of sensorimotor contingency” fits well as an aspect of the present concept of quasi-depiction; it could be proposed that one part of quasi-depicting one's environment is precisely to make predictions of how the bottom-up sensory input from this environment will change as new motor actions are performed. However, O'Regan and Noë (2001) did not take the model-based, heterophenomenological approach that has been adopted here.

## 7. Understanding “qualia:” why do we describe our conscious experience the ways we do?

One objection that could be raised against all of the theories of consciousness discussed above, including the one being put forth here, is that they are *physicalist* (Stoljar, 2009) theories, which some would say concern themselves too unilaterally with third-person observations of overt reports and brain activity, and too little with *subjective, first-person experience* (Nagel, 1974; Jackson, 1982; Chalmers, 1995; Block, 2009): “So what,” one might reason, “if your NCCs or your heterophenomenological theory can predict exactly under what circumstances I will tend to say that I am having what conscious experiences, I want to understand why I have any experiences *at all*, and why they feel the way they do *to me*!”

A possible approach to these matters is to take the narrative behavior model, imagine an organism functioning in accordance with it, and discuss what such an organism, assuming that it has language abilities, would have to say about its own first-person experience. To begin with, consider this organism engaging in a rich variety of behaviors, both overt and covert. Some of these behaviors may be more or less obviously innate to the organism, for example breathing, moving an arm to reach an object, or the covert narrative behavior of quasi-depicting a specific hue of green. Other behaviors may be more obviously learned behaviors, such as for example whistling, or generating the covert quasi-description LION for a certain class of visual stimuli.

For all of these behaviors, whether covert or overt, innate or learned, there are a number of things which can happen in conjunction with them. For instance, by means of mechanisms for familiarity or episodic memory, the organism may *recognize that it has engaged in similar behavior before*. As has been discussed above, another effect of similarity across multiple occasions (e.g., repeatedly seeing lions) is that it shapes the narrative behavior pyramid, by establishment of new quasi-descriptions in the functional hierarchy, with a one-to-many association to nodes at lower levels. In practice, this amounts to a form of *labeling of the organism's own behaviors*, which as discussed previously could occur either together with learning of actual words in a shared language, or without any such association.

The other side of similarity, recognition, and labeling, is *discrimination of differences*, for example between two quasi-descriptions LION and CAT. This ability of discrimination is present also at lower, quasi-depictive levels, for example as discrimination between examples within a quasi-description: discrimination between two different lions, two different hues of green, two different ways of reaching for an object.

In sum, the narrative behavior machinery is capable of creating narratives about the fact that the organism is doing *something*, whether this is something that the organism has done *before*, when it is *different* from another something, and the narrative behavior machinery also puts different *labels* on different somethings, which may or may not be associated with words in a language. In general, however, the machinery does not have any privileged access to *what, in more detail*, the organism is doing, or *how* it is doing it^3^.

**Figure 5:**
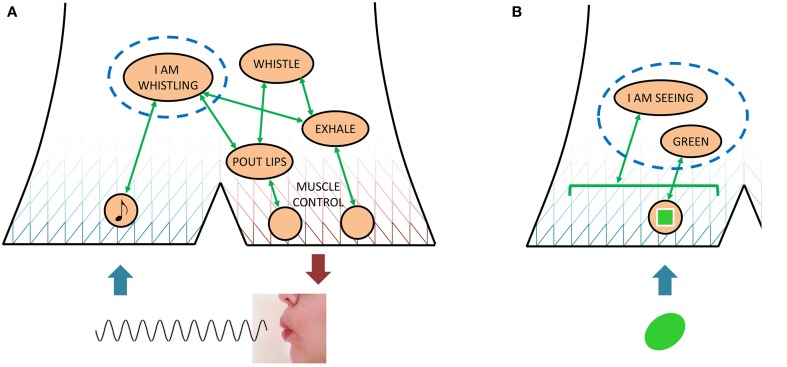
**An illustration of how the narrative behavior model describes a judgment of whether or not one is engaged in a certain behavior, be it an overt one such as whistling (A) or a covert one such as seeing a green color (B)**. In both cases, what is required for a positive judgment is sufficient activation of those covert behaviors that associate with the quasi-description in question (I AM WHISTLING and I AM SEEING GREEN, respectively). However, only in the overt case are there also externally observable components and results of behavior, which permit the behaving individual to construct a coarse explanation of *how* it is achieving the behavior in question.

However, for overt behaviors, which by definition cause externally observable effects, the organism will generally be able to provide at least cursory answers also to the *what in detail* and *how* questions. Consider, again, the example of whistling: Once the organism can say of itself “I am whistling” (i.e., once it has successfully activated a quasi-description I AM WHISTLING), it can proceed to observe its own lip positioning, tongue movements, and exhalation, and since there are commonly agreed words for all of these things in the language it shares with other individuals, the organism can construct an explanation of what it is doing when it is whistling (and such explanation from others may also have been part of how the organism acquired the whistling skill to begin with). However, for successful whistling performance the organism still need not be anywhere near a detailed description of the shape of its lip aperture, exactly where it is putting its tongue, how much air pressure it is applying, and definitely not of what brain structures and neurons are involved. If the organism is particularly inquisitive and motivated, and has access to appropriate observational technology, it can certainly proceed toward such descriptive ability. If the organism does so, it will in the end (since, **ex hypothesi**, it functions according to the narrative behavior model) find that what is required for itself to admit to being whistling is a certain pattern of neuronal activation (I AM WHISTLING), which in turn is activated by a constrained set of other activation patterns, presumably related to quasi-depictions of tones and of the involved overt, muscular activations; see Figure 5A.

Now, compare the above to a situation where the organism could say of itself “I am seeing green,” illustrated in Figure 5B. The organism could mount a similar research project to understand this phenomenon, with a very similar final conclusion; a certain pattern of neuronal activation (I AM SEEING GREEN) being activated by a constrained set of other activation patterns, in this case quasi-depictions of the color green. However, due to the lack of externally observable effects such as lip movements and exhalations, this latter research project would have to start at a much more scientifically advanced level, involving light of a certain frequency and receptors in the retina. Therefore, if the organism does *not* undertake and complete these two research projects, the organism could come to think rather differently of seeing green than of whistling. With whistling, as with overt behavior in general, the organism knows when it is doing it, and may feel that it has a rather good grasp of how it is doing it (even though in fact it ignores most of what is going on). With seeing green, as with covert behavior in general, the organism also knows when it is doing it, but it is more or less clueless as to how. For this reason, the organism might not think about covert narrative behaviors, such as those involved in “seeing green,” as behavior at all, not at all as “doing” something, but may instead use other words, for example “perception,” “subjective experience,” or “subjective state,” to describe “seeing green” and other phenomena that only involve covert behaviors.

Lacking the third-party perspective of a scientifically advanced empirical research project, the organism can nevertheless, from within its own first-person perspective, reach some conclusions about its “perception” or “subjective state,” i.e., about its own covert narrative behaviors. For example, the organism can pick up on the converging nature of the narrative behavior hierarchy. Since it is capable of discriminating between, say, different hues of green, the organism can detect that the overt statement “I am seeing green” is a very crude approximation of what it is actually “perceiving:” The organism can discriminate between when I AM SEEING GREEN(*i*) is active, as opposed to I AM SEEING GREEN(*i* + 1), as well as between individual and even more specific quasi-depictions, and it can therefore conclude that there are details regarding its “subjective state” which are “private,” as in available to itself, from its own first-person perspective, but not to the third-person perspective of other individuals, and “ineffable,” as in not possible to fully communicate to other individuals.

Furthermore, a certain green color will trigger a very specific complex of quasi-depictions and quasi-descriptions, with associated activations of for example color-specific predictions of how the narratives will change with motor activity (i.e., sensorimotor contingencies), or weak activations of color-specific episodic memories, which might in turn evoke subtle color-specific emotional responses. The specificity of this compound reaction is detectable to the organism from its first-person perspective, in the sense that the organism can react with familiarity to the entire complex; the organism can note that all the “somethings” come in their usual grouping, i.e., I AM SEEING GREEN(*i*) is accompanied by the same quasi-depictions, diffuse emotional responses etc., as usual. When the organism detects this specificity, it could, if it thought of its perception as behavior, make a statement like “I am doing something very specific when this green color is presented to me.” However, without this perspective, the organism might rather say “I see this green color in a very specific way” or “there is something very specific which it is like for me to see this color,” and, if the organism is of the philosophically inclined sort, maybe also things like “I will call the specific way I see this green color a quale, and use the word qualia to speak, in general, of the specific ways I see things, hear things, smell things, etc.”

A final characteristic of the narrative behavior machinery that the organism could surmise is that it is really something quite astonishing. The “seeing green” example above, although complex in its own right, is simplistic compared to what the organism is otherwise doing all the time, every day: Continuously constructing, completely without words in a language, an extremely rich, multi-level narrative about what is going on inside and outside of its body, and all of this without the slightest bit of insight for the organism into how it is being achieved, just *that* it is. The situation can to some extent be compared to that of overlearned overt behaviors that have been acquired mainly by practice, like for example whistling or riding a bike, where one can, if one thinks about it, marvel at being capable of doing something without really understanding how. This is an appropriate type of awe for the narrative behavior organism: “Astonishingly, from my first-person perspective I know that I am continuously constructing an extremely rich covert narrative about what it is like to be me, but I do not know at all how I am doing it.” However, without having made the connection between perception and behavior, instead relying on concepts like “qualia” or “my private, ineffable, subjective experience,” the organism may instead formulate the same sense of awe thus: “Astonishingly, from my first-person perspective I know that I have an extremely rich, continuous, subjective experience of what it is like to be me, but I do not know at all what this subjective experience is or how it arises.”

From our third-person perspective, we can see how this way of talking sets the organism up for confusion. It is now, vaguely, discussing its “subjective experience” as something that one “has,” something that “is” something, or something that “arises,” all implying that the “subjective experience” *exists* in some sense. This is not necessarily incorrect as such, but what is not at all clear from the organism's choice of words is that if its “subjective experience” exists, it is in the same way as for example “biking” exists: as a *class of behavior*, where each candidate instance of behavior can be judged by an observer as belonging to the class or not. This is a special kind of emergent existence, different (at least in degree) from how physical objects, like for example the body or the bike, exist, and in this sense the organism would not be entirely mistaken if it considered its “subjective experience” to exist in a non-physical way, or felt unsure regarding its physicality. An impression of non-physicality could be further bolstered by the fact that in contrast with “biking,” the “subjective experience” is without direct traces in the external, physical world, and detectable only to the organism itself. One can therefore not blame the organism if it meets with incredulity the statement that this seemingly non-physical “subjective experience” is, deep down, just patterns of neuronal activations, and expresses this incredulity as there being a fundamentally hard problem of consciousness to be solved (Chalmers, 1995), or an explanatory gap to be bridged: “the experience of green is a subjective state, but brain states are objective, and we do not understand how a subjective state could be an objective state or even how a subjective state could be based in an objective state” (Block, 2009, p. 1113, with credit to Nagel, 1974). From our third-person perspective, however, we see that a “subjective state” arising for the narrative behavior organism, out of its “objective state” of neuronal activity, is, while astonishing and scientifically challenging in many ways, not considerably more so than “a state of biking” arising out of neuronal activity.

In sum, it has been argued here that if an organism that functions in accordance with the narrative behavior model tries to describe those characteristics of its own covert narrative behavior that it can detect from a first-person perspective, it could come up with concepts like “qualia” or “subjective experience,” and be quite perplexed by them. To the extent that the narrative behavior model is a close enough account of what is going on in a human brain, the argument above could hold also for explaining the human tendency for such concepts and perplexedness. Partially related arguments have been made before (Dehaene and Naccache, 2001; Dennett, 2001, O'Regan and Noë, 2001), but the narrative behavior model provides additional structure to the reasoning.

## 8. Conclusion and outlook

Of what has been said here, two points stand out as the most important:

1. The suggestion that instead of assuming that there are general and identifiable neural correlates of consciousness at *T*_c_, the putative moment of conscious experience, (what has here been referred to as *the NCC assumption*), any general common denominator is instead to be sought at *T*_j_, the moment of judging the conscious experience. Specifically, it has been suggested that an individual will admit to a conscious experience of *X* at time *T*_x_ ≈ *T*_c_ if at time *T*_j_ the individual finds itself acceptably capable of covert narrative behavior describing *X*, with increases in *T*_j_ − *T*_x_ probably leading to lowered thresholds for what is acceptable, and to changes in what brain mechanisms are involved in the judgment. Note that this point does not hinge upon the view of perception as behavior; the previous sentence could just as well be reformulated as “if at time *T*_j_ the individual finds that it can establish an acceptable perceptual representation of *X*.”
2. The suggestion that talking about perception as behavior, i.e., *not* replacing “covert narrative behavior about X” with “perceptual representation of X” above, can be helpful in dispelling some of the sense of mystery surrounding consciousness. Perhaps the main benefit of this change of semantics is that it casts perception as one region within a spectrum of differences in degree, rather than as a fundamentally distinct phenomenon. On this view, humans engage in narrative behavior describing what it is like to be themselves along a full range from overt narratives in a shared language, via language-like covert narratives where the units of description are not words in a language, but comparable to such (here referred to as quasi-description), all the way to covert narratives where the unit of description is more specific, for example comparable to fragments of a picture (here referred to as quasi-depiction). Furthermore, it can help to think of a human detecting that it is engaged in covert narrative behavior as doing something very similar to detecting that it is engaged in an overt behavior, in both cases with equally limited ability of determining, from its first-person perspective, *how* it is performing these behaviors.

These two main points have been supported by a number of auxiliary arguments, all of which could individually be further developed: The proposed similarity in neural mechanisms between perception and action selection could be investigated in more detail, perhaps starting from existing theories of brain function which emphasize the question of how perceptual and motor systems relate to each other (e.g., Cisek, 2007; Adams et al., 2013). Note, however, that the main point (2) above does not depend on perception and action selection being supported by *exactly* the same mechanisms. If they were, it would be very clear that we ought to refer to the two with one single term, for example “behavior”. If the two are instead just roughly similar (which seems more probable), we can absorb perception into the term behavior and use this way of talking as a possible handle on consciousness, all the while acknowledging that covert and overt behavior are slightly different types of behavior, just as one might discern subtypes of overt behavior, with differing neural underpinnings.

Furthermore, the narrative behavior model introduced to make point (1) above had to cover a very wide range of phenomena, from low-level sensory processing to language; basically all of the so called “easy problems” of consciousness (Chalmers, 1995). Consequently it has been necessary to keep this model rather simplistic and approximate. In its current boxes-and-arrows form it nevertheless has many similarities to existing, quantitatively defined models (Dehaene et al., 2003; Zylberberg et al., 2010). Such quantification and simulation could allow a more exact specification of the model, as well as a more rigorous test of another supporting argument; that the narrative behavior model is compatible with all of the three experiments considered in detail here, to a greater extent than for example the global neuronal workspace and higher-order theories.

This empirical grounding can also be further extended, by considering additional experiments from the literature, or carrying out new ones. One possible direction would be to study neural events not only at *T*_c_ ≈ *T*_x_, but also close to the time *T*_q_ of requesting the actual subjective report. The account proposed here suggests that at *T*_q_, subjects will try to recreate covert narrative behaviors that occurred in response to the stimulus, and if so the neural signatures of their doing so should correlate better with the actual report than what any neural signatures at *T*_c_ ≈ *T*_x_ do. Especially so if subjects do not know beforehand what stimulus they will be questioned about (as in the experiment by Pitts et al., 2012), such that the actual judgment occurs at *T*_j_ ≈ *T*_q_ rather than at *T*_j_ ≈ *T*_x_, but it is possible that the predicted effect could exist also in the latter case, if the introspective judgment persists all the way up to *T*_q_. Another line of inquiry has already been hinted at above: At modifications of a contrastive paradigm, for example with respect to the time *T*_q_ − *T*_x_, or what happens during this time, the present account predicts shifts in what neural signatures at *T*_c_ ≈ *T*_x_ correlate best with subjective report at *T*_q_. Such shifts, for which the experimental data reviewed here provide some support, should be especially notable if the paradigm modifications cause a change in what brain mechanisms are involved in judging the own ability of narrative behavior; from trying to see an ambiguous currently available stimulus (*T*_j_ = *T*_x_), to maintaining or recalling a very recently presented stimulus, to recognizing a stimulus that was presented a longer while ago. But it is also possible that even minor experimental modifications, such as small changes to *T*_q_ − *T*_x_, could engender slight shifts in observed neural correlates. Again, experimental paradigms that do not instruct subjects on exactly what stimuli they will be asked about later could be especially useful.

### Conflict of interest statement

The author declares that the research was conducted in the absence of any commercial or financial relationships that could be construed as a potential conflict of interest.

## Footnotes

^1^In a later paper, Pitts et al. (2004) also argue that the brain activity they observe does not seem consistent with the GNW hypothesis.

^2^Examples of similar formulations from other authors: “The *content of consciousness” refers to the specific information that I am aware of at a given moment*. (Dehaene et al., 2014, p. 76); “[…] the brain basis of the changing content of consciousness […]” (Tononi and Koch, 2008, p. 256); “This has fooled us into thinking that we know what we are conscious of.” (Lamme, 2010, p. 204); “the neural events reflected in the Nd1-Nd2 sequence may be adequate in themselves to produce visual awareness” (Pitts et al., 2012, p. 297). According to the view presented here, these quotes are all examples of a problematic way of talking about consciousness.

^3^In Wittgenstein's (1967) terms, the narrative behavior machinery can state that there is something in the box, and it can choose to call it a beetle, but that is as far as it can go.

## References

[B1] Adams R. A. Shipp S. Friston K. J. (2013). Predictions not commands: active inference in the motor system. Brain Struct. Funct. 218, 611–643. 10.1007/s00429-012-0475-5 23129312 PMC3637647

[B2] Albouy G. Sterpenich V. Balteay E. Vandewalle G. Desseilles M. Dang-Vu T.. (2008). Both the hippocampus and striatum are involved in consolidation of motor sequence memory. Neuron 58, 261–272. 10.1016/j.neuron.2008.02.008 18439410

[B3] Aru J. Bachmann T. Singer W. Melloni L. (2012). Distilling the neural correlates of consciousness. Neurosci. Biobehav. Rev. 36, 737–746. 10.1016/j.neubiorev.2011.12.003 22192881

[B4] Ashby F. G. (2013). Categorization, neural basis, in The Encyclopedia of the Mind, ed Pashler H. (Thousand Oaks, CA: Sage Publishing), 130–134.

[B5] Ashby F. G. Turner B. O. Horitz J. C. (2010). Cortical and basal ganglia contributions to habit learning and automaticity. Trends Cogn. Sci. 14, 208–215. 10.1016/j.tics.2010.02.001 20207189 PMC2862890

[B6] Baars B. J. (1988). A Cognitive Theory of Consciousness. New York, NY: Cambridge University Press.

[B7] Badre D. D'Esposito M. (2009). Is the rostro-caudal axis of the frontal lobe hierarchical? Nat. Rev. Neurosci. 10, 659–669. 10.1038/nrn2667 19672274 PMC3258028

[B8] Bear M. F. Connors B. W. Paradiso M. A. (2001). Neuroscience: Exploring the Brain. Baltimore, MD: Lippincott Williams & Wilkins.

[B9] Bichot N. P. Rossi A. F. Desimone R. (2005). Parallel and serial neural mechanisms for visual search in macaque area V4. Science 308, 529–534. 10.1126/science.1109676 15845848

[B10] Blackmore S. (2012). Turning on the light to see how the darkness looks, in Consciousness: Its Nature and Functions, eds Kreitler S. Maimon O. (New York, NY: Nova), 109–124.

[B11] Blackmore S. (2015). The neural correlates of consciousness, in This Idea Must Die, ed Brockman J. (New York, NY: HarperCollins), 141–144.

[B12] Block N. (2009). Comparing the major theories of consciousness, in The Cognitive Neurosciences IV, Chap. 77, ed Gazzaniga M. (Cambridge, MA: MIT Press), 1111–1122.

[B13] Cantwell G. Crossley M. W. Ashby F. G. (2015). Multiple stages of learning in perceptual categorization: evidence and neurocomputational theory. Psychon. Bull. Rev. [Epub ahead of print]. 10.3758/s13423-015-0827-2 25917141 PMC4624621

[B14] Carruthers P. (2011). Higher-order theories of consciousness, in The Stanford Encyclopedia of Philosophy, Fall 2011 Edn., ed Zalta E. N. Available online at: http://plato.stanford.edu/archives/fall2011/entries/consciousness-higher/ (Accessed March 3, 2015).

[B15] Chalmers D. (1995). Facing up to the problem of consciousness. J. Conscious. Stud. 2, 200–219.

[B16] Cisek P. (2007). Cortical mechanisms of action selection: the affordance competition hypothesis. Philos. Trans. R. Soc. B 362, 1585–1599. 10.1098/rstb.2007.2054 17428779 PMC2440773

[B17] Cohen M. A. Dennett D. C. (2011). Consciousness cannot be separated from function. Trends Cogn. Sci. 15, 358–364. 10.1016/j.tics.2011.06.008 21807333

[B18] Cooper R. Shallice T. (2000). Contention scheduling and the control of routine activities. Cogn. Neuropsychol. 17, 297–338. 10.1080/026432900380427 20945185

[B19] Cooper R. P. Shallice T. (2006). Hierarchical schemas and goals in the control of sequential behavior. Psychol. Rev. 113, 887–916. 10.1037/0033-295X.113.4.887 17014307

[B20] Crump M. J. C. Logan G. D. (2010). Hierarchical control and skilled typing: evidence for word-level control over the execution of individual keystrokes. J. Exp. Psychol. Learn. Mem. Cogn. 36, 1369–1380. 10.1037/a0020696 20919783

[B21] Curtis C. E. D'Esposito M. (2003). Persistent activity in the prefrontal cortex during working memory. Trends Cogn. Sci. 7, 415–423. 10.1016/S1364-6613(03)00197-9 12963473

[B22] de Graaf T. A. de Jong M. C. Goebel R. van Ee R. Sack A. T. (2011). On the functional relevance of frontal cortex for passive and voluntarily controlled bistable vision. Cereb. Cortex 21, 2322–2331. 10.1093/cercor/bhr015 21385836

[B23] de Graaf T. A. Hsieh P.-J. Sack A. T. (2012). The ‘correlates’ in neural correlates of consciousness. Neurosci. Biobehav. Rev. 36, 191–197. 10.1016/j.neubiorev.2011.05.012 21651927

[B24] De Weerd P. Gattass R. Desimone R. Ungerleider L. G. (1995). Responses of cells in monkey visual cortex during perceptual filling-in of an artificial scotoma. Nature 377, 731–734. 10.1038/377731a0 7477262

[B25] Deco G. Rolls E. T. (2004). A neurodynamical cortical model of visual attention and invariant object recognition. Vision Res. 44, 621–642. 10.1016/j.visres.2003.09.037 14693189

[B26] Deco G. Rolls E. T. (2005). Attention, short-term memory, and action selection: a unifying theory. Prog. Neurobiol. 76, 236–256. 10.1016/j.pneurobio.2005.08.004 16257103

[B27] Dehaene S. Changeux J.-P. Naccache L. Sackur J. Sergent C. (2006). Conscious, preconscious, and subliminal processing: a testable taxonomy. Trends Cogn. Sci. 10, 204–211. 10.1016/j.tics.2006.03.007 16603406

[B28] Dehaene S. Charles L. King J.-R. Marti S. (2014). Toward a computational theory of conscious processing. Curr. Opin. Neurobiol. 25, 76–84. 10.1016/j.conb.2013.12.005 24709604 PMC5635963

[B29] Dehaene S. Naccache L. (2001). Towards a cognitive neuroscience of consciousness: basic evidence and a workspace framework. Cognition 79, 1–37. 10.1016/S0010-0277(00)00123-2 11164022

[B30] Dehaene S. Sergent C. Changeux J.-P. (2003). A neuronal network model linking subjective reports and objective physiological data during conscious perception. Proc. Natl. Acad. Sci. U.S.A. 100, 8520–8525. 10.1073/pnas.1332574100 12829797 PMC166261

[B31] Dennett D. C. (1978). Toward a cognitive theory of consciousness, in Perception and Cognition: Issues in the Foundations of Psychology, Minnesota Studies in the Philosophy of Science, Vol. IX, ed Wade Savage C. (Minneapolis, MN: University of Minnesota Press), 201–228.

[B32] Dennett D. C. (1988). Quining qualia, in Consciousness in Contemporary Science, eds Marcel A. J. Bisiach E. (Oxford: Oxford University Press), 42–77.

[B33] Dennett D. C. (1991). Consciousness Explained. Boston, MA: Little, Brown and Co.

[B34] Dennett D. C. (1992). Filling in versus finding out: a ubiquitous confusion in cognitive science, in Cognition, Conception, and Methodological Issues, eds Pick H. L. Jr. van Den Broek P. Knill D. C. (Washington, DC: American Psychological Assocation), 33–49.

[B35] Dennett D. C. (2001). Are we explaining consciousness yet? Cognition 79, 221–237. 10.1016/S0010-0277(00)00130-X 11164029

[B36] Desimone R. Duncan J. (1995). Neural mechanisms of selective visual attention. Annu. Rev. Neurosci. 18, 193–222. 10.1146/annurev.ne.18.030195.001205 7605061

[B37] Di Lollo V. von Mühlenen A. Enns J. T. Bridgeman B. (2004). Decoupling stimulus duration from brightness in metacontrast masking: data and models. J. Exp. Psychol. Hum. Percept. Perform. 30, 733–745. 10.1037/0096-1523.30.4.733 15301621

[B38] Dux P. E. Marois R. (2009). The attentional blink: a review of data and theory. Atten. Percept. Psychophys. 71, 1683–1700. 10.3758/APP.71.8.1683 19933555 PMC2915904

[B39] Engström J. (2008). A Model of Attention Selection in Driving. Licentiate thesis, Chalmers University of Technology, Sweden.

[B40] Feldman J. A. (2006). From Molecule to Metaphor: A Neural Theory of Language. Cambridge, MA: The MIT Press.

[B41] Feredoes E. Heinen K. Weiskopf N. Ruff C. Driver J. (2011). Causal evidence for frontal involvement in memory target maintenance by posterior brain areas during distracter interference of visual working memory. Proc. Natl. Acad. Sci. U.S.A. 108, 17510–17515. 10.1073/pnas.1106439108 21987824 PMC3198359

[B42] Fuster J. M. (2000). Executive frontal functions. Exp. Brain Res. 133, 66–70. 10.1007/s002210000401 10933211

[B43] Goldberg A. E. (2003). Constructions: a new theoretical approach to language. Trends Cogn. Sci. 7, 219–224. 10.1016/S1364-6613(03)00080-9 12757824

[B44] Graziano M. S. A. Aflalo T. N. (2007). Mapping behavioral repertoire onto the cortex. Neuron 56, 239–251. 10.1016/j.neuron.2007.09.013 17964243

[B45] Graziano M. S. A. Aflalo T. N. Cooke D. F. (2005). Arm movements evoked by electrical stimulation in the motor cortex of monkeys. J. Neurophysiol. 94, 4209–4223. 10.1152/jn.01303.2004 16120657

[B46] Gurney K. N. Humphries M. D. Redgrave P. (2015). A new framework for cortico-striatal plasticity: Behavioural theory meets *in vitro* data at the reinforcement-action interface. PLoS Biol. 13:e1002034. 10.1371/journal.pbio.1002034 25562526 PMC4285402

[B47] Harrison S. A. Tong F. (2009). Decoding reveals the contents of visual working memory in early visual areas. Nature 458, 632–635. 10.1038/nature07832 19225460 PMC2709809

[B48] Henke K. (2010). A model for memory systems based on processing modes rather than consciousness. Nat. Rev. Neurosci. 11, 523–532. 10.1038/nrn2850 20531422

[B49] Ishai A. Ungerleider L. G. Haxby J. V. (2000). Distributed neural systems for the generation of visual images. Neuron 28, 979–990. 10.1016/S0896-6273(00)00168-9 11163281

[B50] Jackson F. (1982). Epiphenomenal qualia. Philos. Q. 32, 127–136. 10.2307/29600779854266

[B51] Jannati A. Di Lollo V. (2012). Relative blindsight arises from a criterion confound in metacontrast masking: Implications for theories of consciousness. Conscious. Cogn. 21, 307–314. 10.1016/j.concog.2011.10.003 22051554

[B52] Johnson J. S. Spencer J. P. Schöner G. (2008). Moving to higher ground: the dynamic field theory and the dynamics of visual cognition. N. Ideas Psychol. 26, 227–251. 10.1016/j.newideapsych.2007.07.007 19173013 PMC2630705

[B53] Kastner S. De Weerd P. Desimone R. Ungerleider L. G. (1998). Mechanisms of directed attention in the human extrastriate cortex as revealed by functional MRI. Science 282, 108–111. 10.1126/science.282.5386.108 9756472

[B54] Koivisto M. Revonsuo A. (2010). Event-related brain potential correlates of visual awareness. Neurosci. Biobehav. Rev. 34, 922–934. 10.1016/j.neubiorev.2009.12.002 20005249

[B55] Kosslyn S. M. (2005). Mental images and the brain. Cogn. Neuropsychol. 22, 333–347. 10.1080/02643290442000130 21038254

[B56] Lakoff G. (1987). Women, Fire, and Dangerous Things. Chicago, IL: The University of Chicago Press.

[B57] Lamme V. A. F. (2006). Towards a true neural stance on consciousness. Trends Cogn. Sci. 10, 494–501. 10.1016/j.tics.2006.09.001 16997611

[B58] Lamme V. A. F. (2010). How neuroscience will change our view on consciousness. Cogn. Neurosci. 1, 204–240. 10.1080/17588921003731586 24168336

[B59] Latash L. P. Latash M. L. (1994). A new book by N. A. Bernstein: “On dexterity and its development.” J. Mot. Behav. 26, 56–61. 10.1080/00222895.1994.9941662 15757835

[B60] Lau H. Rosenthal D. (2011). Empirical support for higher-order theories of conscious awareness. Trends Cogn. Sci. 15, 365–373. 10.1016/j.tics.2011.05.009 21737339

[B61] Lau H. C. Passingham R. E. (2006). Relative blindsight in normal observers and the neural correlate of visual consciousness. Proc. Natl. Acad. Sci. U.S.A. 103, 18763–18768. 10.1073/pnas.0607716103 17124173 PMC1693736

[B62] Leopold D. A. Logothetis N. K. (1996). Activity changes in early visual cortex reflect monkeys' percepts during binocular rivalry. Nature 379, 549–553. 10.1038/379549a0 8596635

[B63] Levine J. (1983). Materialism and qualia: the explanatory gap. Pac. Philos. Q. 64, 354–361. 23073546

[B64] Li Q. Hill Z. He B. J. (2014). Spatiotemporal dissociation of brain activity underlying subjective awareness, objective performance and confidence. J. Neurosci. 34, 4382–4395. 10.1523/JNEUROSCI.1820-13.2014 24647958 PMC3960476

[B65] Marti S. Sigman M. Dehaene S. (2012). A shared cortical bottleneck underlying attentional blink and psychological refractory period. NeuroImage 59, 2883–2898. 10.1016/j.neuroimage.2011.09.063 21988891

[B66] Miller E. K. Cohen J. D. (2001). An integrative theory of prefrontal cortex function. Annu. Rev. Neurosci. 24, 167–202. 10.1146/annurev.neuro.24.1.167 11283309

[B67] Nagel T. (1974). What is it like to be a bat? Philos. Rev. 83, 435–450. 10.2307/2183914

[B68] Norman D. Shallice T. (1986). Attention to action: willed and automatic control of behavior, in Consciousness and Self Regulation, Vol. 4, chap. 1, eds Davidson R. Schwartz G. Shapiro D. (New York, NY: Plenum), 1–18.

[B69] O'Regan J. K. Noë A. (2001). A sensorimotor account of vision and visual consciousness. Behav. Brain Sci. 24, 939–1031. 10.1017/S0140525X01000115 12239892

[B70] Pastor-Bernier A. Cisek P. (2011). Neural correlates of biased competition in premotor cortex. J. Neurosci. 31, 7083–7088. 10.1523/JNEUROSCI.5681-10.2011 21562270 PMC6703218

[B71] Pessoa L. Thompson E. Noë A. (1998). Finding out about filling-in: a guide to perceptual completion for visual science and the philosophy of perception. Behav. Brain Sci. 21, 723–802. 10.1017/S0140525X98001757 10191878

[B72] Pierce W. D. Cheney C. D. (2004). Behavior Analysis and Learning, 3rd Edn. Mahwah, NJ: Lawrence Erlbaum Associates.

[B73] Pitts M. A. Martínez A. Hillyard S. A. (2012). Visual processing of contour patterns under conditions of inattentional blindness. J. Cogn. Neurosci. 24, 287–303. 10.1162/jocn_a_00111 21812561

[B74] Pitts M. A. Padwal J. Fennelly D. Martínez A. Hillyard S. A. (2014). Gamma band activity and the p3 reflect post-perceptual processes, not visual awareness. NeuroImage 101, 337–350. 10.1016/j.neuroimage.2014.07.024 25063731 PMC4169212

[B75] Porcu E. Keitel C. Müller M. M. (2014). Visual, auditory and tactile stimuli compete for early sensory processing capacities within but not between senses. NeuroImage 97, 224–235. 10.1016/j.neuroimage.2014.04.024 24736186

[B76] Postle B. R. (2006). Working memory as an emergent property of the mind and brain. Neuroscience 139, 23–38. 10.1016/j.neuroscience.2005.06.005 16324795 PMC1428794

[B77] Raz A. Lamar M. Buhle J. T. Kane M. J. Peterson B. S. (2007). Selective biasing of a specific bistable-figure percept involves fMRI signal changes in frontostriatal circuit: a step toward unlocking the neural correlates of top-down control and self-regulation. Am. J. Clin. Hypn. 50, 137–156. 10.1080/00029157.2007.10401611 18030926 PMC2386759

[B78] Rohrer T. (2006). Image schemata in the brain, in From Perception to Meaning: Image Schemas in Cognitive Linguistics, ed Hampe B. (Berlin: Mouton de Gruyter), 165–196.

[B79] Rounis E. Maniscalco B. Rothwell J. C. Passingham R. E. Lau H. (2010). Theta-burst transcranial magnetic stimulation to the prefrontal cortex impairs metacognitive visual awareness. Cogn. Neurosci. 1, 165–175. 10.1080/17588921003632529 24168333

[B80] Schendan H. E. Searl M. M. Melrose R. J. Stern C. E. (2003). An fMRI study of the role of the medial temporal lobe in implicit and explicit sequence learning. Neuron 37, 1013–1025. 10.1016/S0896-6273(03)00123-5 12670429

[B81] Schlegel A. Kohler P. J. Fogelson S. V. Alexander P. Konuthula D. Tse P. U. (2013). Network structure and dynamics of the mental workspace. Proc. Natl. Acad. Sci. U.S.A. 110, 16277–16282. 10.1073/pnas.1311149110 24043842 PMC3791746

[B82] Schöner G. Kopecz K. Erlhagen W. (1997). The dynamic neural field theory of motor programming: Arm and eye movements, in Self-Organization, Computational Maps, and Motor Control, eds Morasso P. Sanguineti V. (New York, NY: Elsevier Science B. V.), 271–310.

[B83] Scott M. Yeung H. H. Gick B. Werker J. F. (2013). Inner speech captures the perception of external speech. J. Acoust. Soc. Am. 133, EL286–EL292. 10.1121/1.4794932 23556693

[B84] Seger C. A. Miller E. K. (2010). Category learning in the brain. Annu. Rev. Neurosci. 33, 203–219. 10.1146/annurev.neuro.051508.135546 20572771 PMC3709834

[B85] Sergent C. Baillet S. Dehaene S. (2005). Timing of the brain events underlying access to consciousness during the attentional blink. Nat. Neurosci. 8, 1391–1400. 10.1038/nn1549 16158062

[B86] Skinner B. F. (1984). Behaviorism at fifty. Behav. Brain Sci. 7, 615–667. 10.1017/S0140525X00027618

[B87] Srinivasan R. Russel D. P. Edelman G. M. Tononi G. (1999). Increased synchronization of neuromagnetic responses during conscious perception. J. Neurosci. 19, 5435–5448. 10377353 10.1523/JNEUROSCI.19-13-05435.1999 PMC6782339

[B88] Sterzer P. Haynes J.-D. Rees G. (2006). Primary visual cortex activation on the path of apparent motion is mediated by feedback from hMT+/V5. NeuroImage 32, 1308–1316. 10.1016/j.neuroimage.2006.05.029 16822682

[B89] Stoljar D. (2009). Physicalism, in The Stanford Encyclopedia of Philosophy, Fall 2009 Edn., Zalta E. N. Available online at: http://plato.stanford.edu/archives/fall2009/entries/physicalism/ (Accessed March 16, 2015).

[B90] Tononi G. (2004). An information integration theory of consciousness. BMC Neurosci. 5:42. 10.1186/1471-2202-5-42 15522121 PMC543470

[B91] Tononi G. (2012). Integrated information theory of consciousness: an updated account. Arch. Ital. Biol. 150, 290–326. 10.4449/aib.v149i5.1388 23165867

[B92] Tononi G. Koch C. (2008). The neural correlates of consciousness: an update. Ann. N.Y. Acad. Sci. 1124, 239–261. 10.1196/annals.1440.004 18400934

[B93] Uithol S. van Rooij I. Bekkering H. Haselager P. (2012). Hierarchies in action and motor control. J. Cogn. Neurosci. 24, 1077–1086. 10.1162/jocn_a_00204 22288396

[B94] von der Heydt R. Friedman H. S. Zhou H. (2003). Searching for the neural mechanism of color filling-in, in Filling-In: From Perceptual Completion to Cortical Reorganization, chap. 6, eds Pessoa L. de Weerd P. (Oxford: Oxford University Press), 106–127.

[B95] Wittgenstein L. (1967). Philosophical Investigations, 3rd Edn. Oxford: Basil Blackwell.

[B96] Yin H. H. Ostlund S. B. Balleine B. W. (2008). Reward-guided learning beyond dopamine in the nucleus accumbens: the integrative functions of cortico-basal ganglia networks. Eur. J. Neurosci. 28, 1437–1448. 10.1111/j.1460-9568.2008.06422.x 18793321 PMC2756656

[B97] Yonelinas A. P. Otten L. J. Shaw K. N. Rugg M. D. (2005). Separating the brain regions involved in recollection and familiarity in recognition memory. J. Neurosci. 25, 3002–3008. 10.1523/JNEUROSCI.5295-04.2005 15772360 PMC6725129

[B98] Zylberberg A. Fernández Slezak D. Roelfsema P. R. Dehaene S. Sigman M. (2010). The brain's router: a cortical network model of serial processing in the primate brain. PLoS Comput. Biol. 6:e1000765. 20442869 10.1371/journal.pcbi.1000765 PMC2861701

